# Itavastatin and resveratrol increase triosephosphate isomerase protein in a newly identified variant of TPI deficiency

**DOI:** 10.1242/dmm.049261

**Published:** 2022-05-17

**Authors:** Andrew P. VanDemark, Stacy L. Hrizo, Samantha L. Eicher, Jules Kowalski, Tracey D. Myers, Megan R. Pfeifer, Kacie N. Riley, Dwight D. Koeberl, Michael J. Palladino

**Affiliations:** 1 Biological Sciences and Structural Biology, University of Pittsburgh, Pittsburgh, PA 15260, USA; 2 Department of Pharmacology and Chemical Biology, University of Pittsburgh School of Medicine, Pittsburgh, PA 15261, USA; 3 Pittsburgh Institute for Neurodegenerative Diseases, University of Pittsburgh School of Medicine, Pittsburgh, PA 15261, USA; 4 Department of Biology, Slippery Rock University of Pennsylvania, Slippery Rock, PA 16057, USA; 5 Department of Pediatrics, Division of Medical Genetics, Duke University Medical Center, Durham, NC 27710, USA

**Keywords:** Biochemistry, Glycolytic dysfunction, Structural biology, Triosephosphate isomerase

## Abstract

Triosephosphate isomerase (TPI) deficiency (TPI Df) is an untreatable glycolytic enzymopathy that results in hemolytic anemia, progressive muscular impairment and irreversible brain damage. Although there is a ‘common’ mutation (*TPI^E105D^*), other pathogenic mutations have been described. We identified patients who were compound heterozygous for a newly described mutation, *TPI^Q181P^*, and the common *TPI^E105D^* mutation. Intriguingly, these patients lacked neuropathy or cognitive impairment. We then initiated biochemical and structural studies of TPI^Q181P^. Surprisingly, we found that purified TPI^Q181P^ protein had markedly impaired catalytic properties whereas crystallographic studies demonstrated that the TPI^Q181P^ mutation resulted in a highly disordered catalytic lid. We propose that genetic complementation occurs between the two alleles, one with little activity (*TPI^Q181P^*) and one with low stability (*TPI^E105D^*). Consistent with this, *TPI^Q181P/E105D^* fibroblasts exhibit a significant reduction in the TPI protein. These data suggest that impaired stability, and not catalytic activity, is a better predictor of TPI Df severity. Lastly, we tested two recently discovered chemical modulators of mutant TPI stability, itavastatin and resveratrol, and observed a significant increase in TPI in *TPI^Q181P/E105D^* patient cells.

## Abstract (precis)

**Summary:** A newly identified triosephosphate isomerase (TPI) variant (TPI^Q181P^) confers TPI deficiency, suggests a molecular and genetic model for its pathogenesis, and the potential for therapeutic intervention.

## INTRODUCTION

Triosephosphate isomerase deficiency (TPI Df) is a severe genetic metabolic disorder that presents early on in childhood. Typically, TPI Df patients are born healthy but hemolytic anemia leads to recurrent infections. The patient experiences developmental delays and a rapidly progressive disease course with symptoms related to muscle weakness and neurological impairment. Muscle weakness and wasting impairs locomotor function (limb and core muscles) as well as breathing in some cases (diaphragm). The disease typically progresses further resulting in severe and irreversible neurologic pathology and early death. Currently, there are no drug treatments available for TPI Df. Patients are provided support for their symptoms, often with diet and nutritional support that is of unknown clinical value. Thus, the prognosis remains grim, and few patients survive to adulthood.

TPI Df can be caused by a handful of pathogenic missense mutations that result in specific amino acid substitutions in TPI, the most common of which is *TPI^E105D^*. Numerous TPI Df-causing pathogenic mutations have been studied and, thus far, pathogenic mutations encode proteins that retain at least some function, but typically are unstable leading to protein degradation and lowered TPI levels. We have identified a novel TPI Df variant, *TPI^Q181P^*, that has an atypical and less severe disease course. The mutation was found in two compound heterozygous sibling patients with the common mutation. The *TPI^Q181P/E105D^* patients we observed had severe skeletal muscle symptoms; however, their anemia was relatively mild and their cognitive function remained normal even into adulthood.

TPI is a homodimer with each monomer adopting the (βα)_8_ TIM barrel fold (Banner et al., 1972; Lolis et al., 1990). Access to the catalytic site is restricted by the catalytic lid which is formed by loop 6. The catalytic lid is dynamic and has been observed in both the open and closed states (Wierenga et al., 1992). Transitions between the open and closed state of the catalytic lid are coordinated with changes in the positions of prominent residues within the active site including E165, K13, H95 and S96 (Massi et al., 2006; Wierenga et al., 1992; Zhang et al., 1994). Numerous structures have been solved for wild-type and mutant TPI, including several disease-associated mutant proteins (Roland et al., 2015; Roland et al., 2019). TPI^E105D^ exhibits altered solvation and reduced stability of the TPI dimer (Rodriguez-Almazan et al., 2008), whereas the catalytic lid and active site residues within the TPI^I170V^ mutant resemble the closed state (Roland et al., 2015). Recently, we have reported the structure of the TPI^R190Q^ mutant, in which the mutation is positioned distant from the active site. The mutation of R190 results in the loss of two salt bridge interactions that are critical for protein stability. The loss of these interactions also conferred an altered arrangement of catalytic site residues (Roland et al., 2019). Overall, these studies have highlighted the tight coordination between the active site and the conformation and dynamics of the catalytic lid.

To date, animal studies of TPI in the mouse have not effectively modeled TPI Df (Conway et al., 2018; Pretsch, 2009; Segal et al., 2019), and most studies of pathogenesis have been conducted in the fruit fly *Drosophila* (Celotto et al., 2006; Hrizo et al., 2013; Hrizo and Palladino, 2010; Roland et al., 2013, 2016; Seigle et al., 2008). One particularly informative mouse study concluded that a TPI mutation that does not grossly impair TPI stability, even though it significantly impairs its catalytic function, fails to result in neuromuscular impairment, and thus does not model TPI Df well (Segal et al., 2019). *Drosophila* mutants with a unique mutation known as *TPI^sugarkill^* (*TPI^sgk^*) appear to model neuromuscular aspects of the disease well, and thus were the first model organism for this disease (Celotto et al., 2006). One study with *TPI^sgk^* mutants generated compound heterozygotes with a catalytically inactive allele, *TPI^sgk/deltaCat^*, and the animals were surprisingly less severely affected when compared with homozygous *TPI^sgk^* animals (Roland et al., 2013). Although the animals still had neuromuscular impairment, locomotor function was significantly improved and the compound heterozygous animals did live significantly longer (Roland et al., 2013). This was the first report of genetic complementation between two *TPI* alleles, albeit incomplete complementation. The conclusion was that TPI^sgk^ retained significant amounts of activity but the protein itself was unstable and rapidly degraded, whereas TPI^deltaCat^ was inactive but relatively stable. Thus, the compound heterozygotes were less affected due to partial genetic complementation between these alleles.

Although there is some variability in the TPI Df disease course that is not completely understood, there is a prevalence of evidence on numerous pathogenic mutations that supports the conclusions that (1) pathogenic variants retain significant activity, (2) TPI Df mutant proteins exhibit reduced stability presumably due to accelerated turnover by protein quality control (PQC) machinery, and (3) impaired stability and not activity correlates the most with disease severity. As such, we and others have proposed that impairing mutant TPI turnover is the most promising avenue for the development of TPI Df treatments (Hrizo et al., 2021; Segal et al., 2019; Vogt et al., 2021). Recent studies have utilized RNA interference (RNAi) screens to identify potential therapeutic targets (Hrizo et al., 2021) and exploited human cellular models to develop high-throughput optical screens to enable direct drug screening for novel TPI Df therapeutics (Vogt et al., 2021). Interestingly, these studies identified two compounds, resveratrol and itavastatin, that increase the levels of the common mutant TPI^E105D^ protein in a human cellular TPI Df model and also in TPI Df patient cells (Vogt et al., 2021). Here, we report that these compounds also increase protein levels in *TPI^Q181P/E105D^* patient fibroblasts, suggesting that these compounds should be tested for efficacy in animal models as they would represent a first-ever treatment for TPI Df. Importantly, the *in vitro* patient cellular data suggest that these compounds could benefit not only patients with the common mutation but compound heterozygous patients such as those reported here.

## RESULTS

### Clinical descriptions

Two siblings with childhood-onset locomotor impairment were identified and evaluated clinically for metabolic disease and possible TPI Df.

#### Patient 1

A 21-year-old female with a history of developmental delay, spastic diplegia, and cerebellar ataxia was seen. The patient was the product of a full-term, normal vaginal delivery with no complications. She was developing normally until the age of 10 months, when development seemed to regress after a fall resulting in injury to her head. The parents reported she was back on track by 12 months and walked at approximately 18 months, but with a noticeably abnormal gait and upper body posture. Dystonia and tightness of her left side, as well as a tremor in her hands, were observed around the same time. Gross motor delays persisted, and the patient continued to have spasticity and dystonic posturing in the lower extremities, as well as slurring of her speech.

As part of her work-up, she had a thorough evaluation that included muscle biopsy and oxidative phosphorylation studies in 2007, which identified a mitochondrial complex I deficiency, although no molecular genetic etiology was identified. She had been placed on coenzyme Q10 and reported that she did not notice any changes when she was taking it.

On review of systems, the patient denied anemia, pallor, jaundice (other than during the newborn age), muscle atrophy or seizures. She did acknowledge fatigue, spasms, intention tremor and dystonia. The patient receives Botox for tightness in her neck and left arm and also feels tightness in her shoulders. She reported being on continuous positive airway pressure (CPAP) at night for both muscle weakness and central apnea. Cognitive function was normal although speech was hard to understand due to hyper-nasality. The patient communicated well using assistive devices and appeared to be at age level for reading and comprehension.

#### Patient 2

The patient was a 17-year-old male with a history of dystonia, cerebellar ataxia, speech delay, unsteadiness, tremor and hypotonia and was patient 1's sibling. He presented in early childhood with ataxia, tremors and dystonia. Patient 2's early development was mostly on track, although his parents reported that he sat a little late, but are not sure of the exact age. He was walking between 13 and 14 months, but reportedly had an abnormal gait, similar to his sister, and seemed to use posturing to maintain balance. Patient 2 spoke his first words around 18 months of age and received speech therapy. He made fast progress in speech per report.

Like his sister, patient 2 received a diagnosis of mitochondrial complex I deficiency based on reduced activity identified by electron transport chain testing completed in 2007, but no nuclear or mitochondrial gene mutations were identified as etiology for the complex I deficiency.

On review of systems, patient 2 and his parents denied anemia, pallor, dystonia, seizures and cardiomyopathy. They acknowledged some fatigue, intention tremor and spasms. They noted that he has muscle atrophy and explained that because his feet were ‘turning in’, he had a surgical correction that made it difficult to stand or walk (his family attributed his muscle atrophy in the calves and thighs to difficulty standing or walking following this procedure). His past medical history is also notable for surgery at 15 years of age due to his lower jaw protruding. His family explained that he uses a Trilogy ventilator at night due to muscle weakness and central apnea. As with patient 1, cognitive function appeared normal, but speech suffered from hyper-nasality and the patient communicated well with assistive devices. The patient appeared to be at age level for reading and comprehension.

### Mutational analyses

**Fig. 1.**
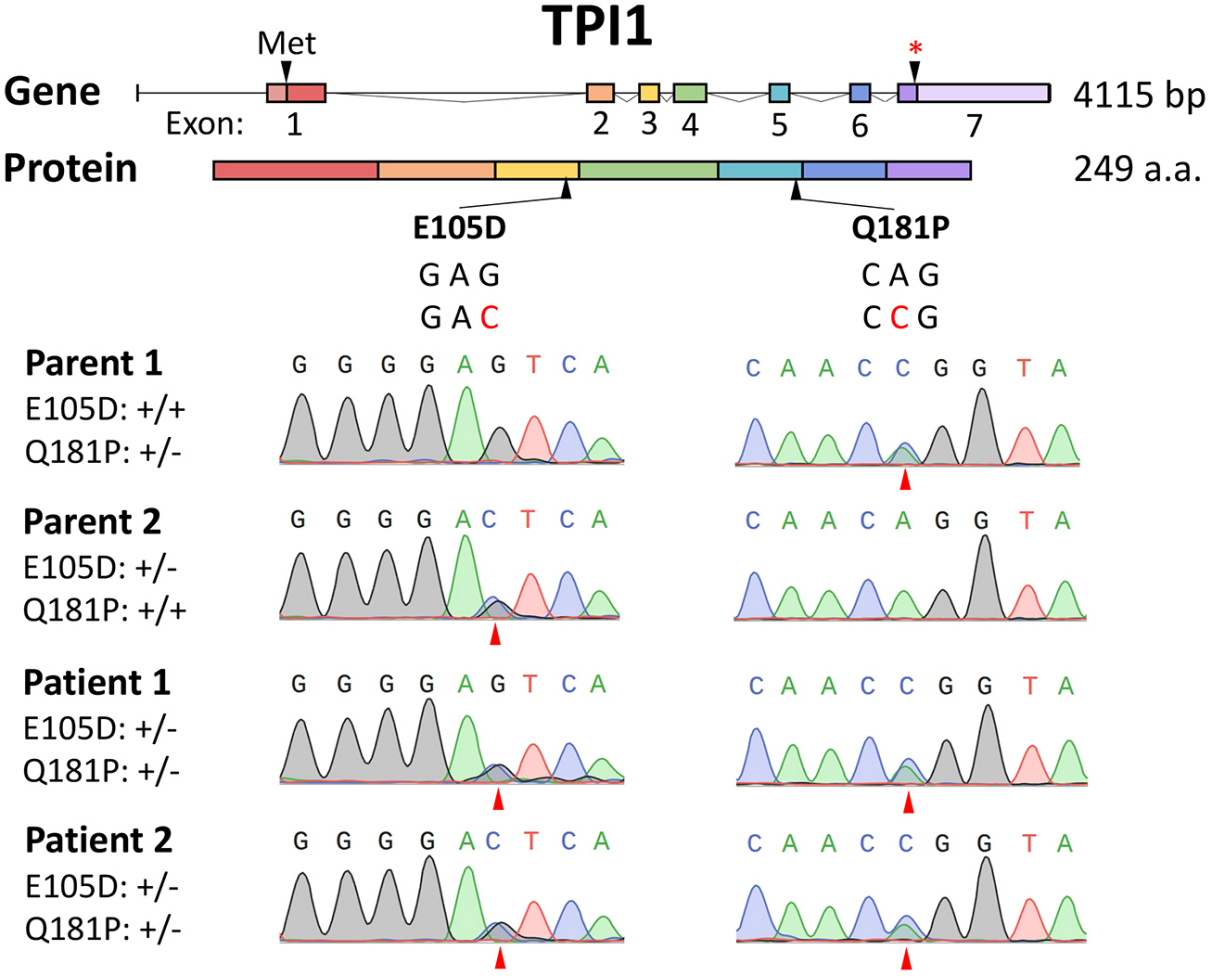
**Sequence analysis of the *TPI* locus.** (A) Illustration of the *TPI* gene showing the location of each mutation. (B) Chromatographic data demonstrating the mutations observed and confirming both patients are compound heterozygotes for the Q181P and E105D mutations.

Both patients were found to be compound heterozygous for two *TPI* variants (*c.315G>C; p.E105D* and *c.542A>C; p.Q181P*) via whole-exome sequencing done at GeneDx in 2014. The *TPI^E105D^* variant is seen in about 80% of variant alleles in patients with clinical TPI deficiency, and *TPI^Q181P^* is classified as a variant of uncertain significance. Patient fibroblasts were obtained from both the patients and parents, and the *TPI* locus was PCR amplified and coding regions were sequenced. These studies confirmed that *TPI^Q181P^* and *TPI^E105D^* are the only mutations present, that both patients are compound heterozygotes for these alleles and that the *TPI^E105D^* variant was paternally inherited (parent 2) whereas the *TPI^Q181P^* was inherited maternally (parent 1; Fig. 1). Although the adenine to cytosine transversion is a missense mutation resulting in a Q181P amino acid substitution, it also affects the −2 position relative to the exon 5 splice donor in pre-mRNA. Reverse transcription PCR (RT-PCR) was performed and exon skipping was not evident, consistent with the expression of TPI^Q181P^ protein (Fig. S1).

### TPI^Q181P^ protein biochemistry

**Fig. 2.**
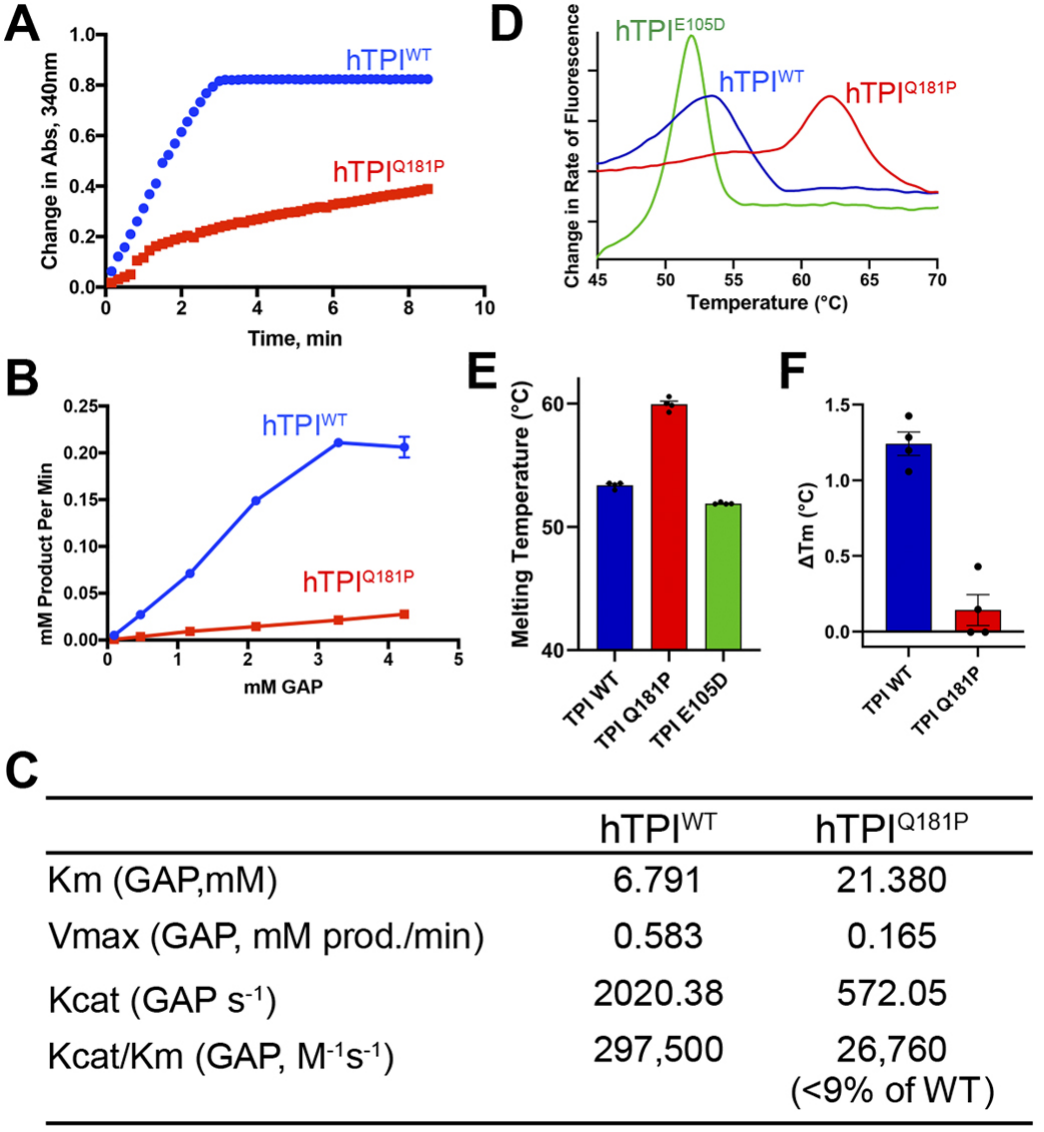
**TPI^Q181P^ exhibits reduced substrate binding and rate of catalysis compared with the wild-type enzyme.** (A) TPI activity from a spectrophotometric enzyme-linked assay of 1 ng purified human TPI (hTPI) enzyme in the reaction with 2.115 mM of GAP. Abs, absorbance. (B) The absorbance data were used to determine the amount (mM) of product formed per minute by 1 ng of TPI enzyme at varying concentrations of the substrate GAP. The average for each concentration of GAP is shown, *n*=3. (C) Prism-calculated V_max_, K_m_ and K_cat_. (D) Representative curves from differential scanning fluorimetry (DSF) showing increased thermal stability for TPI^Q181P^ (red) as compared with TPI^WT^ (blue) and TPI^E105D^ (green). (E) Quantification of DSF from WT, Q181P and E105D TPI variants (*n*=4). Error bars represent s.e.m. *P*<0.00001 (two-sample one-tailed unpaired *t-*test). (F) Change in T_m_ from DSF measurements of TPI^WT^ and TPI^Q181P^ with the addition of 10 mM DHAP. Error bars represent s.e.m. (*n*=4), one-tailed unpaired *t*-test was used to determine significance. *P*<0.00001.

**Fig. 3.**
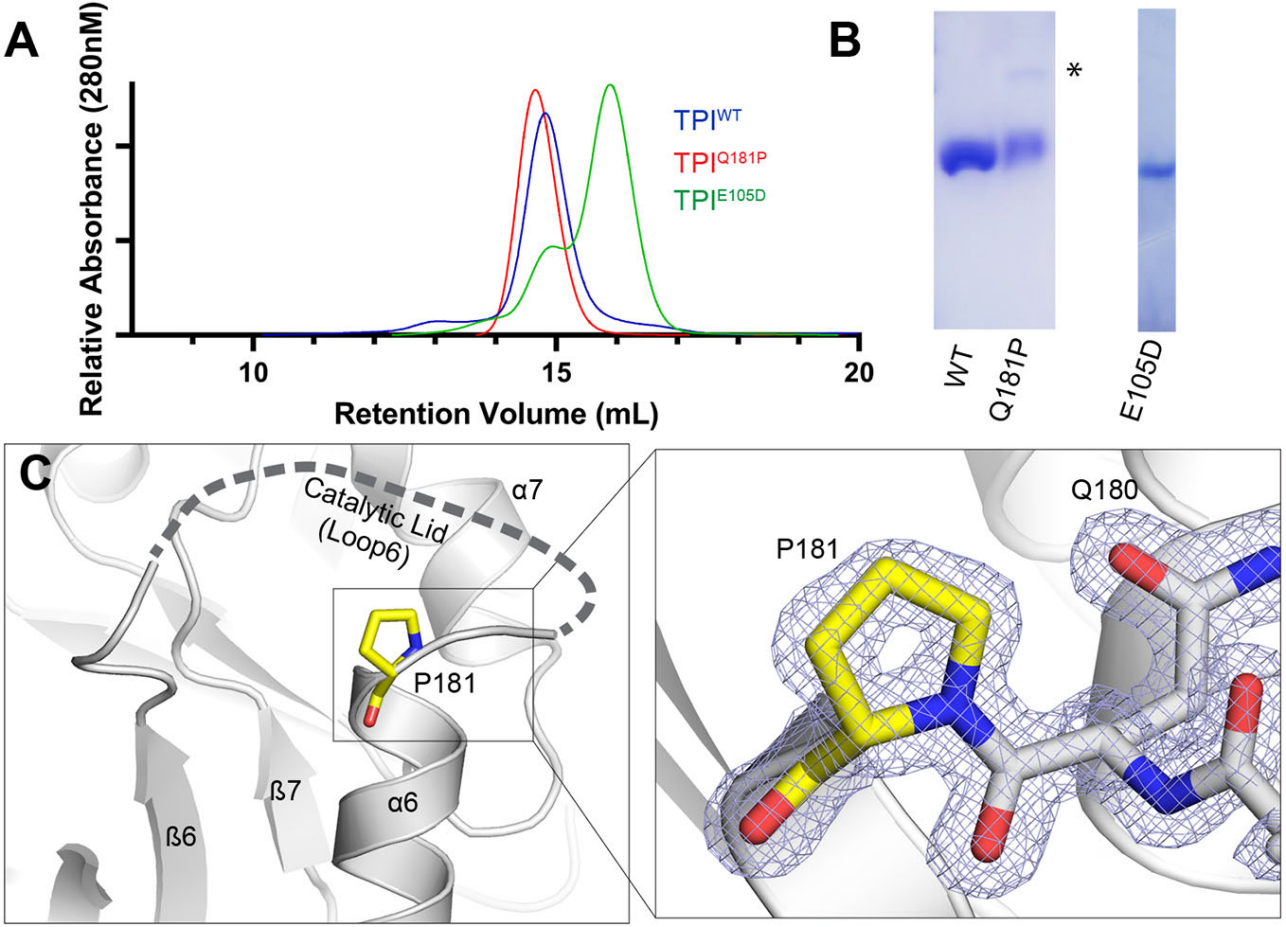
**Biophysical and structural characterization of TPI^Q181P^.** (A) Size exclusion chromatography demonstrating that both TPI^WT^ and TPI^Q181P^ proteins are dimeric and show no gross changes in overall structure or stoichiometry. TPI^E105D^ appears to exist in a monomer-dimer equilibrium under these conditions. (B) Coomassie Blue staining of the purified TPI variants used in this study. Asterisk indicates a minor contaminant from purification. (C) The catalytic lid region from the structure of TPI^Q181P^. Electron density for P181 is shown (right), however the catalytic lid is largely disordered.

To better understand the significance of the *TPI^Q181P^* mutation, recombinant human TPI^Q181P^ and TPI^wild-type^ (TPI^WT^) proteins were purified for biochemical studies as described previously (Fig. 3B) (Roland et al., 2019). TPI activity was measured using a well-established NADH-linked assay (Roland et al., 2013; Williams et al., 1999). When the enzyme activity of TPI^Q181P^ was directly compared with the activity of TPI^WT^, a significant reduction in both the maximum reaction rate (V_max_) and turnover number (K_cat_) were observed (Fig. 2). These studies demonstrate that the TPI^Q181P^ enzyme has less than 9% of the catalytic turnover rate (K_cat_/K_m_) of the TPI^WT^ enzyme. These are significant changes in enzyme function in comparison to TPI with the E105D ‘common’ mutation, in which the TPI^E105D^ homodimer exhibited 75-97% activity by K_cat_/K_m_ measurements compared with the wild-type enzyme in three different published studies (Cabrera et al., 2018; De La Mora-De La Mora et al., 2013; Rodriguez-Almazan et al., 2008). The changes observed in TPI^181P^ enzyme activity are more similar to the activity observed for TPI^G72A^ and TPI^V231M^, which exhibited ∼23% and ∼11.2% activity compared with TPI^WT^ by K_cat_/K_m_ respectively (Cabrera et al., 2018). The Michaelis constant (K_m_) of the TPI^Q181P^ mutant enzyme also significantly increased ∼3-fold over TPI^WT^ indicating that TPI^Q181P^ exhibits reduced substrate binding affinity compared with the wild-type enzyme. The more common TPI^E105D^ mutant exhibited no significant reported change in K_m_ in two separate studies and a less than 2-fold increase over TPI^WT^ in a third study (Cabrera et al., 2018; De La Mora-De La Mora et al., 2013; Rodriguez-Almazan et al., 2008). However, again the TPI^G72A^ and TPI^V213M^ mutants exhibited an increase in K_m_ of ∼6.5-fold and ∼8.7-fold, respectively, over TPI^WT^. This suggests the G72A and V231M mutations are more similar to Q181P than the E105D allele in that they both exhibit significant reductions in catalytic activity of TPI coupled with reduced substrate affinity (Cabrera et al., 2018). We also previously examined the thermal stability of TPI^Q181P^ using differential scanning fluorimetry, and found that TPI^Q181P^, TPI^E105D^ and the wild-type control exhibit single-phase non-reversible denaturation as observed for other TPI mutants (Roland et al., 2015). Similar to the TPI Df-causing mutants TPI^R189Q^ and TPI^I170V^, but unlike TPI^E105D^, we observed a significant increase in the thermal stability for TPI^Q181P^ (59.93±0.26°C, expressed as mean±s.e.m.) over the wild-type control (53.37±0.13°C) (Fig. 2) (*n*=4). Further, we observe that incubation with the substrate dihydroxyacetone phosphate (DHAP) results in a stabilization of wild-type TPI as expected, whereas that effect was significantly reduced in TPI^Q181P^ (Fig. 2). This is consistent with a reduced binding affinity in the TPI^Q181P^ variant. Lastly, we tested the stoichiometry of the WT, E105D and Q181P TPI variants by analytical size-exclusion chromatography. We found that TPI^WT^ and TPI^Q181P^ exhibited very similar retention volumes, whereas the TPI^E105D^ variant appeared to be in a monomer-dimer equilibrium (Fig. 3A). Together, these results indicate that the pathogenic defect associated with TPI^Q181P^ is not the result of a loss of protein folding, but is instead a loss of catalytic function.

### Structure of TPI^Q181P^

In an effort to reveal the molecular defect associated with the Q181P mutation, we determined the structure of TPI^Q181P^ using X-ray crystallography at 1.3 Å resolution (see Table 1 and Materials and Methods for a complete description of the crystallization and structure determination process). Crystals were obtained in conditions similar to previous TPI structures, belonging to the space group P2_1_2_1_2_1_ and containing one TPI dimer in the asymmetric unit. Comparing the structures of wild-type TPI (Roland et al., 2015) and TPI^Q181P^, we found that they are highly similar overall as expected with an all atom root mean square deviation (r.m.s.d.) of 0.24 Å, demonstrating that the overall fold of TPI was not disturbed by the mutation. We did however find differences in three important regions of the TPI structure. The first is within the catalytic lid, which we found to be disordered within the TPI^Q181P^ structure. We observed this in both subunits of the dimer; however, the affected regions were not identical, with residues 170-177 being disordered in subunit A, whereas residues 172-179 were disordered in subunit B. The electron density was clearer for the P181 residue in subunit A (Fig. 3C, inset), and thus we use this subunit in our figures and description unless otherwise noted. Biologically important TPI residues are contained within the disordered region including I170, which enhances catalysis by excluding water within the active site (Richard et al., 2016; Roland et al., 2015) and when mutated to valine results in TPI Df (Arya et al., 1997). Also affected are residues 173-175 which form the C-terminal hinge and impact catalytic efficiency (Kursula et al., 2004; Sun and Sampson, 1998). Lastly, A178 was disordered in the B subunit. This residue impacts catalytic efficiency and an A178L mutation has been shown to shift TPI into the closed conformation (Alahuhta et al., 2008). Increased mobility of these key residues found to be disordered in the TPI^Q181P^ mutant provides a molecular rationale for the decrease in substrate affinity we observed biochemically (Fig. 2C).

**Table 1.**
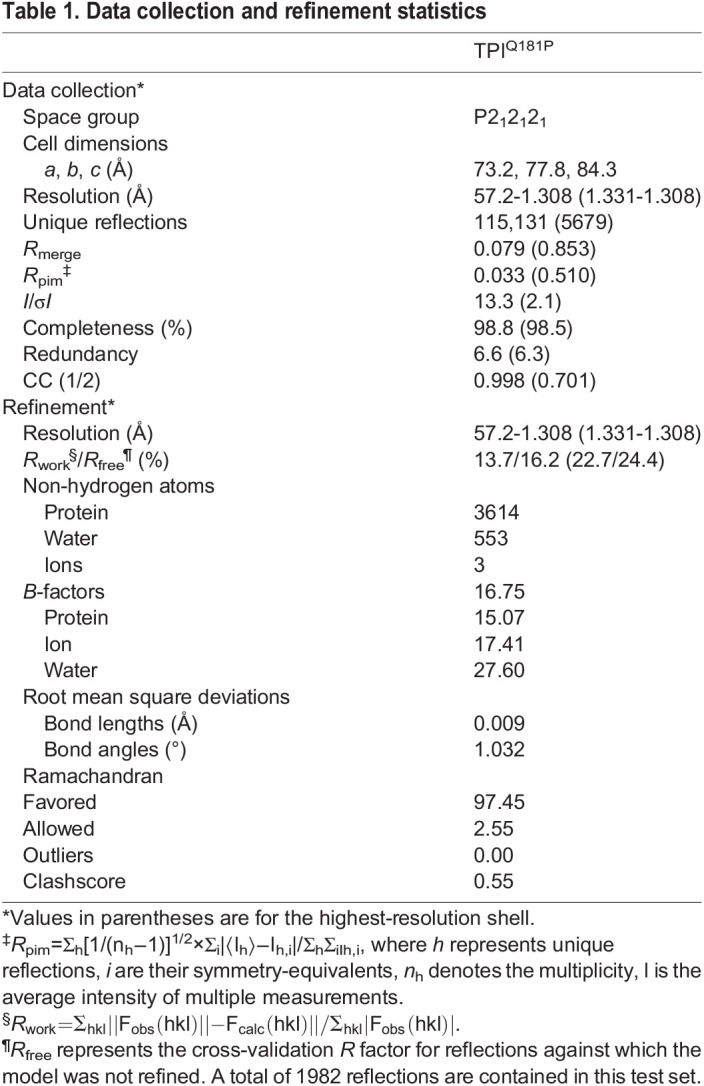
Data collection and refinement statistics

**Fig. 4.**
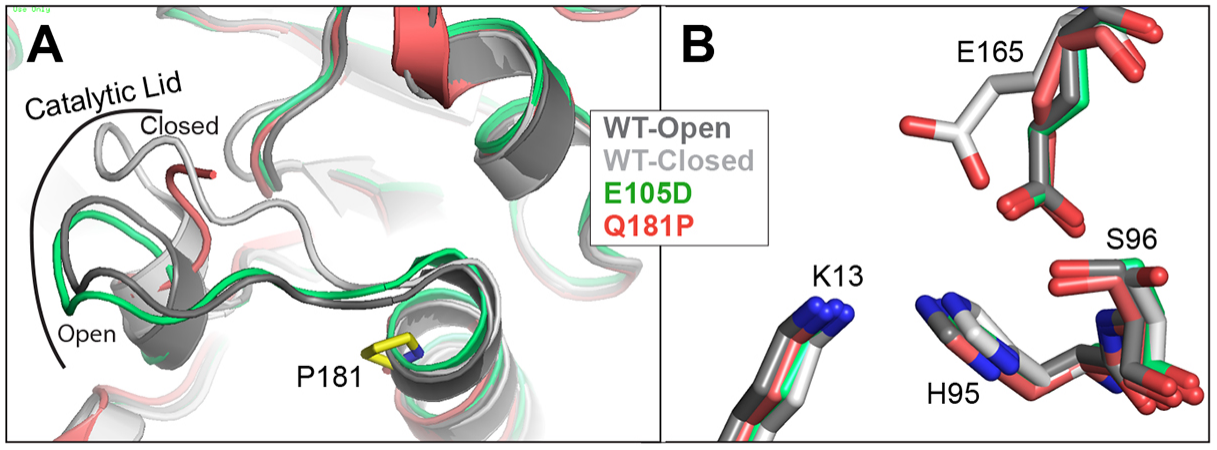
**The Q181P mutation results in structural changes in the catalytic lid.** (A) Superposition of TPI^Q181P^ (red) with TPI^WT^ structures in the open (dark gray) and closed (light gray) states, as well as TPI^E105D^ (green). P181 is highlighted in yellow for reference. (B) The positions of catalytic residues K13, H95, S96 and E165 from the same superposition are shown and colored as in A. The positions of residues E165 and S96 indicate that these residues adopt the open conformation in TPI^Q181P^.

We also observed changes at the C-terminal hinge in the TPI^Q181P^ structure. Prominently, P181 introduces a kink in the main chain at the beginning of helix 6. This disrupts hydrogen bonding interactions that occur between Q181 and T178 in the wild-type structure. These interactions are important as they not only stabilize the beginning of helix 6, but also help to orient residues preceding 178, and thereby directly influence the position of the catalytic lid. A proline at position 181 not only disrupts these interactions but also introduces a kink in the main chain, which prevents residues 177-181 from adopting the helical conformation observed in wild-type TPI structures. Importantly, this disruption is not observed in the structure of the TPI^E105D^ variant (Fig. 4A). Residues 177-181 are observed in a helical conformation in both the open and closed orientation of the catalytic lid, demonstrating that the defect observed is not merely altering the equilibrium between the open and closed states, but is instead grossly affecting the C-terminal hinge and catalytic lid (Fig. 4A).

Lastly, the differences in TPI structure extended into the catalytic site. We crystallized TPI^Q181P^ in the presence of bromide, which we have shown previously to localize in the active site along with a phosphate abstracted from solution. In wild-type TPI, this promotes the closed conformation, with concomitant changes in the key catalytic residues E165 and S96, which are also observed in the TPI^E105D^ variant (Fig. 4A,B). In our TPI^Q181P^ structure, however, we observed only partial occupancy for bromide ions, and no phosphates were observed. Further, we found that residues E165 and S96 adopted the open conformation (Fig. 4B). All of these results point towards a defect in catalytic lid coordination for TPI^Q181P^.

### Mutant TPI protein levels

**Fig. 5.**
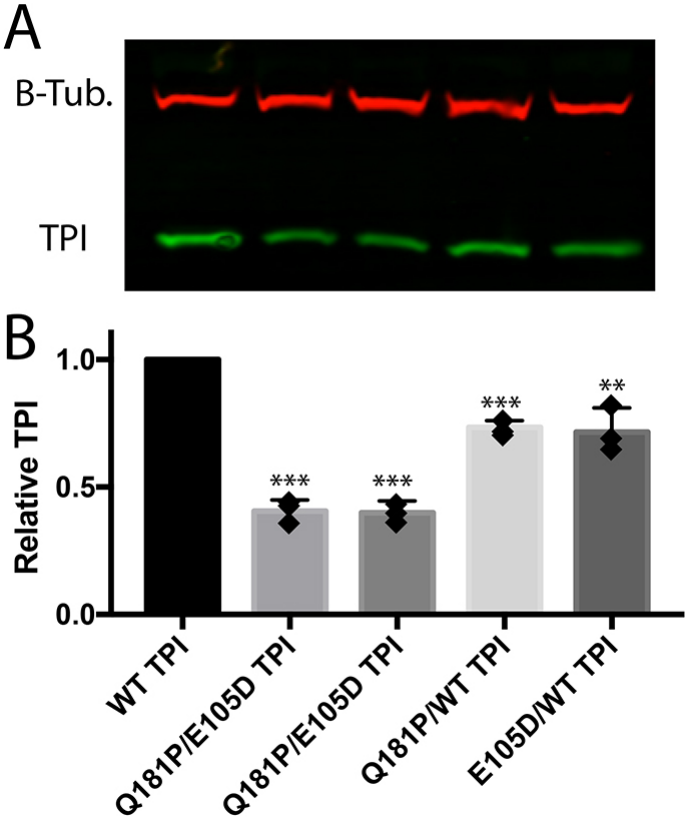
**Western blot data from patient fibroblasts compared with heterozygous unaffected parental controls.** (A,B) Representative western blot (A) of lysates quantified in B. Both patients exhibited ∼40% of normal TPI levels. *n*=3. Two-tailed unpaired Student's *t*-test was used to determine significance. **P<0.005, ***P<0.001, compared with WT. Note: these levels are significantly higher than the ∼10% that was observed in the *TPI^R190Q/E105D^* patient with a much more severe disease course (Roland et al., 2019).

The TPI^Q181P^ protein retains less than 10% of the catalytic activity of the wild-type protein and the molecular defects associated with the Q to P amino acid substitution are consistent with impaired catalysis. However, other mutations in *TPI* that reduce catalysis are not inherently pathogenic unless protein levels are also reduced (Segal et al., 2019). Thus, we examined protein levels in compound heterozygous *TPI^Q181P^*/*TPI^E105D^* patient cells. Western blots using patient fibroblasts demonstrated reduced TPI in patient cells compared with wild-type levels (Fig. 5). Interestingly, both heterozygous *TPI^Q181P^*^/+^ and *TPI^E105D/+^* parent cells exhibit a significant but modest reduction in TPI levels (Fig. 5). These data are consistent with *TPI^Q181P^* being a pathogenic mutation that results in both a decrease in protein activity and overall levels, likely through accelerated turnover of the protein.

### Resveratrol and itavastatin increase TPI levels in *TPI^Q181P/E105D^* cells

**Fig. 6.**
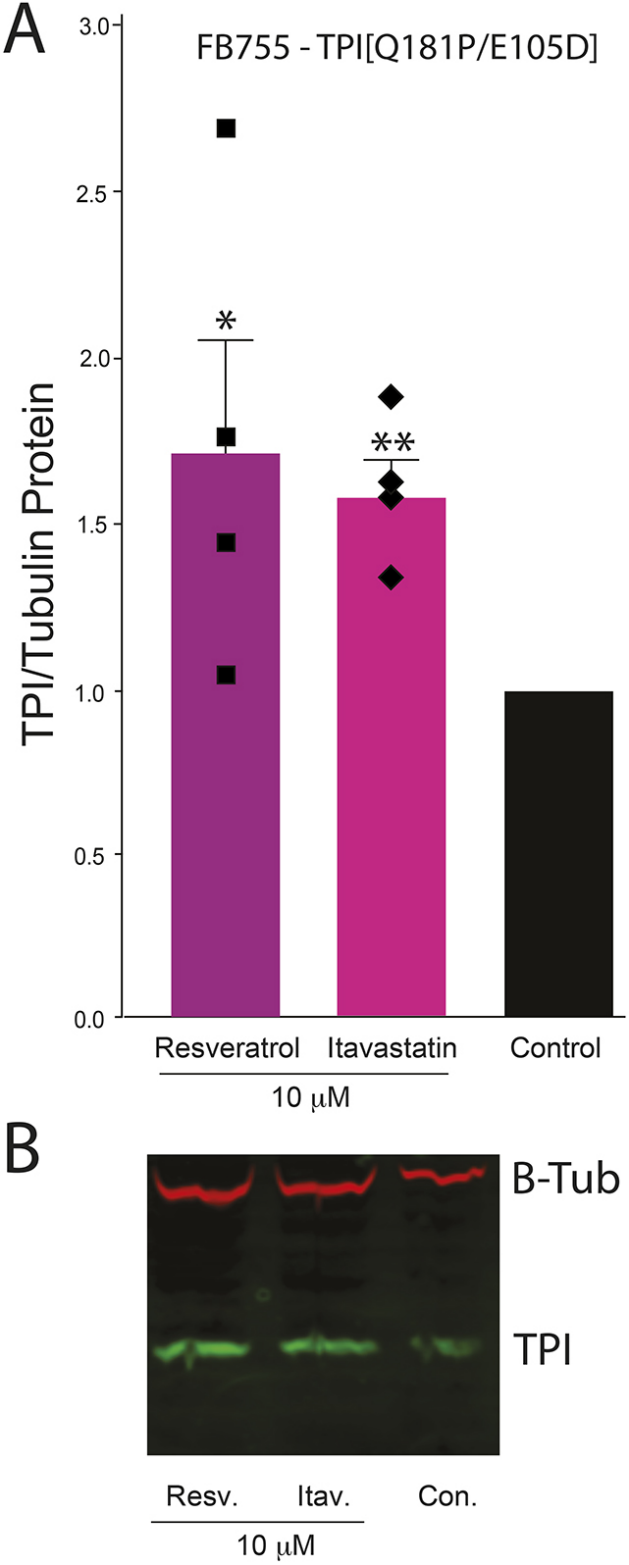
**Resveratrol and itavastatin improve TPI protein levels in *TPI^Q181P/E105D^* patient fibroblasts.** (A) Significant increases in TPI protein levels were observed in patient cells relative to control cells, normalized against total tubulin levels. FB755 indicates TPI^Q181P/E105D^ patient fibroblasts. Values are mean±s.e.m. of four biological replicates. Two-tailed unpaired Student's *t*-test was used to determine significance. *P<0.05; **P<0.005. (B) Representative western blot for the data shown in A. Resv., resveratrol; Itav., itavastatin; Con., control.

Using human embryonic kidney (HEK) cells expressing fluorescently tagged TPI^E105D^ to model TPI Df, we previously screened the NIH clinical collection drug library to identify compounds that increase mutant TPI levels (Vogt et al., 2021). Using this high-throughput assay, resveratrol, a natural supplement, and several statins were identified as potential therapies for TPI Df (Vogt et al., 2021). We asked whether these compounds similarly increase TPI levels in compound heterozygous patient cells. Western blots of *TPI^Q181P^*/*TPI^E105D^* patient cells demonstrate that both resveratrol and itavastatin increase TPI levels by ∼50%, relative to DMSO-treated patient cells (Fig. 6).

## DISCUSSION

*TPI^Q181P^* is a novel *TPI* mutation that results in TPI Df, at least in the *TPI^Q181P^*/*TPI^E105D^* combination observed in these patients. The TPI^Q181P^ protein exhibits a marked reduction in biochemical activity and a significant reduction in the TPI protein was observed in *TPI^Q181P^*/*TPI^E105D^* cells. Certainly, the data are consistent with *TPI^Q181P^* being a pathogenic *TPI* allele capable of causing or contributing to TPI Df. Although the patients were severely affected and the symptoms rapidly progressed, their disease course is less severe than other compound heterozygotes or *TPI^E105D^* homozygotes. Notable is the severity of the muscular symptoms yet the lack of childhood-onset cognitive impairment. This suggests genetic complementation may be occurring between these alleles leading to a reduction in symptoms within parts of the nervous system, similar to what was observed in *TPI^deltaCat/sgk^* animals (Roland et al., 2013).

The severe deficits observed in patients containing *TPI^Q181P^* are mirrored at the molecular level. The high-resolution crystal structure of TPI^Q181P^ shows that this substitution affects both critical active site residues and positioning of the catalytic lid, effectively uncoupling their coordinated movements. This is supported biochemically as well. Further, the increase in K_m_ suggests a loss of substrate-protein interactions, the precise nature of which cannot be observed crystallographically. Ultimately, we show here that the structural changes in the catalytic lid caused by the TPI^Q181P^ mutant correlate with increased rates of protein turnover in cells. It is possible that the enhanced accessibility of residues in the catalytic lid causes TPI^Q181P^ to be recognized by protein quality control machinery, thereby increasing rates of protein turnover.

TPI Df is a severe, rapidly progressive, untreatable disease and there is a pressing need to develop therapies. One approach is to inhibit the proteins that underlie the accelerated turnover of mutant TPI. Genetic screening has found dozens of proteins putatively involved in mutant TPI turnover, suggesting there are numerous possible pharmacological targets (Hrizo et al., 2021). The premise of this approach is that it is likely easier to block degradation of the protein being made by the cell than to correct the dysfunction that results from a severe alteration in intermediate metabolism. TPI is an essential enzyme in organisms from *Escherichia coli* to humans, and the TPI Df pathogenic mutations studied so far encode proteins with greatly reduced activity, but more significantly, have impaired proteolytic cleavage, dimer stability and thermal stability. These findings suggest that increasing the amount of mutant protein may be of therapeutic value, which is supported by results in animal models of TPI Df. Increasing mutant protein, even modestly, using genetic or pharmacological approaches improves longevity and locomotor function (Hrizo and Palladino, 2010; Seigle et al., 2008). Importantly, resveratrol and itavastatin were previously shown to increase mutant TPI protein in *TPI^E105D^* homozygous patient cells, and here we show that they similarly increase mutant protein levels in *TPI^Q181P^/TPI^E105D^* patient cells. Given the presence of the mitochondrial complex I deficiency in the affected siblings described herein and previously described mitochondrial dysfunction in TPI Df flies (Hrizo et al., 2013), resveratrol could be beneficial in TPI Df through its antioxidant effects (de la Lastra and Villegas, 2007). Although these data suggest resveratrol and itavastatin could be of therapeutic value, there remains a pressing need for a mammalian model to test the efficacy *in vivo*. Additionally, although it is likely that increasing the amount of mutant protein will offer immediate benefits in terms of symptomatic relief, the long-term effects of such an approach are not known.

*TPI^E105D^* is the ‘common’ mutation, and it is the only known human *TPI* mutation that results in disease as a homozygote. Numerous patients have been reported that are compound heterozygotes, typically with one of the alleles being *TPI^E105D^*. One such compound heterozygous patient with a *TPI^R190Q^*/*TPI^E105D^* allelic combination, has the most severe presentation that has been reported (Roland et al., 2019). *TPI^Q181P^*/*TPI^E105D^* is the latest compound heterozygous combination reported, and although the disease still presents with significant symptoms, it does appear to be milder, and does not appear to have significant childhood-onset cognitive impairment. It is possible that these alleles genetically complement, particularly in the nervous system, resulting in the less-severe symptoms; however, the basis of this apparent tissue specificity is not understood. Although this is a reasonable hypothesis and is similar to what was observed in *TPI^deltaCat/sgk^* alleles in *Drosophila*, additional studies are needed. Additionally, the patient fibroblast studies presented here are important, but studies with nervous tissues are needed to better elucidate the basis of the apparent genetic complementation.

Pathogenesis in compound heterozygous patients is difficult to study. This is particularly true with a protein that forms a dimer. Theoretically, within *TPI^Q181P^*/*TPI^E105D^* patient cells, TPI^Q181P^-TPI^E105D^ homodimers as well as TPI^Q181P^-TPI^E105D^ heterodimers can form. In fact, there is evidence of heterodimers in a TPI compound heterozygote animal model (Roland et al., 2013). We have measured the biochemical activity of the homodimer protein and determined protein levels in the compound heterozygous patient cells, but a much better understanding of pathogenesis could be achieved with data on the biochemical activity and a structure of the TPI^Q181P^-TPI^E105D^ heterodimer, and with a *TPI^Q181P/E105D^* animal model that would enable direct studies of pathogenesis within neuromuscular tissues.

## MATERIALS AND METHODS

### Human TPI purification and analytical gel filtration chromatography

Coding sequences for wild-type and mutant human TPI (hTPI) were inserted into pLC3 vectors (a generous gift from Graham F. Hatfull, Department of Biological Sciences, University of Pittsburgh) for recombinant expression as a TEV-cleavable His_6_-maltose binding protein (MBP) fusion protein and confirmed by sequencing. Expression was performed in CodonPlus RIPL *E.coli* (Agilent Technologies), using autoinduction media (Studier, 2005) at room temperature for 24 h. Cells were lysed via homogenization in 25 mM Tris-HCl pH 8, 500 mM NaCl, 10% glycerol, 5 mM imidazole and 1mM β-mercaptoethanol (β-ME), and cellular debris removed by centrifugation at 30,000 ***g***. TPI protein was then purified by nickel affinity chromatography, followed by cleavage with TEV protease to remove the His_6_-MBP tag. A second round of nickel affinity purification, followed by anion exchange and gel filtration chromatography were then performed. Peak fractions were concentrated to 6-8 mg/ml prior to crystallization.

Analytical gel filtration chromatography was performed using an analytical S200 (Sigma-Aldrich) at a TPI concentration of 1.5 mg/ml for each TPI variant. Buffering conditions were 20 mM Tris-HCl pH 8, 200 mM NaCl and 1 mM β-ME. Note: The classical convention for numbering TPI amino acids does not include the start methionine. Thus, E105D is also known as E104D in some previously published papers. We have used actual numbering throughout this manuscript as the classical convention complicates structural discussions with a protein that has a start methionine, and also because amino acids 181 and 182 in the human TPI wild-type protein are both glutamine (Q).

### Crystallization and structure determination

Crystals of hTPI^Q181P^ were grown using the sitting-drop vapor-diffusion method at 4°C. The crystals grew overnight in a drop consisting of 1 μl of protein (at a concentration of 6-8 mg/ml) and 2 μl of a well solution containing 14-15% polyethylene glycol (PEG) 2000 monomethyl ether (MME) (Sigma-Aldrich) and 50 mM potassium bromide as in previous structural studies (Roland et al., 2015, 2019, 2013). The crystals were then cryoprotected in 36% PEG 2000 MME and flash frozen in liquid nitrogen prior to X-ray diffraction. Diffraction data were collected at beamline 31-IDD at Argonne National Labs and processed and scaled via autoPROC (Vonrhein et al., 2011) using *I*/σ*I*>2.0 and CC(1/2)>0.3 as cutoffs. Crystals belong to the space group P2_1_2_1_2_1_ with *a*=73.2, *b*=77.8 and *c*=84.3 Å. Phases were estimated using molecular replacement within Phenix (Adams et al., 2010) using TPI^WT^ as a search model (PDB: 4POC) (Roland et al., 2015) and used to calculate an initial map. The initial model was then improved through rounds of model building in Coot (Emsley et al., 2010) and positional and anisotropic B-factor refinement within Phenix. Model quality was assessed using MolProbity within Phenix. Structure factors and atomic coordinates have been deposited into the PDB (www.rcsb.org) under 7RDE.

### Differential scanning fluorimetry

The thermal stability of the hTPI WT and Q181P mutants was determined using differential scanning fluorimetry. TPI^Q181P^ and TPI^WT^ proteins were diluted to 0.1 and 0.25 mg/ml, respectively, in a buffer containing 20 mM HEPES pH 7.5, 15 mM NaCl, 5% glycerol, 1 mM β-ME and 1× Glo-Melt fluorescent dye (Biotium), using the ROX fluorescent dye (Biotium) as a passive reference. Four replicates were performed using QuantStudio 3 Real-Time PCR (Thermo Fisher) and a temperature gradient of 30-95°C. Fluorescence of Glo-Melt was measured at 520 nm, and the melting temperature (T_M_) defined as the peak of the derivative curve. To assess the effect of the TPI^Q181P^ mutant on the ability to interact with substrate, 10 mM DHAP (Sigma-Aldrich) was added into the reaction, and the resulting T_m_ was compared with the same protein in which the same volume of vehicle (water) had been added.

### Protein activity assays

Purified TPI (1 ng) was examined for isomerase activity using a NADH-linked assay previously described (Roland et al., 2013; Williams et al., 1999). Reactions were run in triplicate over a range of 0.095-4.23 mM glyceraldehyde 3-phosphate (GAP; Sigma-Aldrich, St Louis, MO, USA) with an accompanying control without GAP. Absorbance data were collected using a Spectra Max Plus 384 microplate reader (Molecular Devices, Sunnyvale, CA, USA). Enzyme kinetics were evaluated in the initial linear phase of each reaction and were fit to the Michaelis-Menten equation using nonlinear regression in GraphPad V8 Prism (GraphPad Software, La Jolla, CA, USA).

### Culturing patient fibroblasts

Patient and parental fibroblasts were obtained via skin punch (Duke University Health System IRB protocol number 00014158). The cells were de-identified, genotype confirmed and tested for mycoplasma using the Mycofluor Mycoplasma Detection Kit (Thermo Fisher, M7006). Fibroblasts were frequently tested for mycoplasma and were cultured using standard methods (37°C, 5% CO_2_) in complete media [Dulbecco's Modified Eagle Medium (DMEM; Gibco) with 10% fetal bovine serum (FBS; Gibco), 100 U/ml penicillin (Sigma-Aldrich), 100 µg/ml streptomycin (Lonza), 2 mM L–glutamine (Gibco) and supplemental non-essential amino acids (Gibco)].

### Western blotting

Patient and control fibroblast western blotting was performed as previously reported (Vogt et al., 2021). Briefly, cells were trypsinized (using Trypsin 0.05% for 5 min), pelleted, resuspended in RIPA buffer with protease inhibitors [PMSF (100 µM), Leupeptin (1 µg/µl), Pepstatin A (0.5 µg/µl)] and pulse sonicated. BCA assays (Pierce) were used to quantify protein concentrations and whole-cell lysates resolved by SDS-PAGE (12%) were transferred onto 0.45 µm PVDF membranes for immunoblotting using anti-TPI (1:5000; rabbit polyclonal FL-249; Santa Cruz Biotechnology, sc-30145) or anti-β-tubulin (1:1000; mouse polyclonal E7-C; Developmental Studies Hybridoma Bank) diluted in Odyssey Blocking Buffer (Licor). Scanned images were quantified digitally (Image Studio Ver 5.2 software), TPI levels were normalized to the loading control, and differences in TPI expression were evaluated by a two-tailed unpaired Student's *t*-test.

## Supplementary Material

Supplementary information
