# Supplementary material for: Itavastatin and resveratrol increase triosephosphate isomerase protein in a newly identified variant of TPI deficiency
Source: Dis Model Mech. 2022 May 17;15(5):dmm049261. doi: 10.1242/dmm.049261 (PMC9150114; doi:10.1242/dmm.049261)
Supplement: Supplementary information [file dmm-15-049261-s1.pdf]

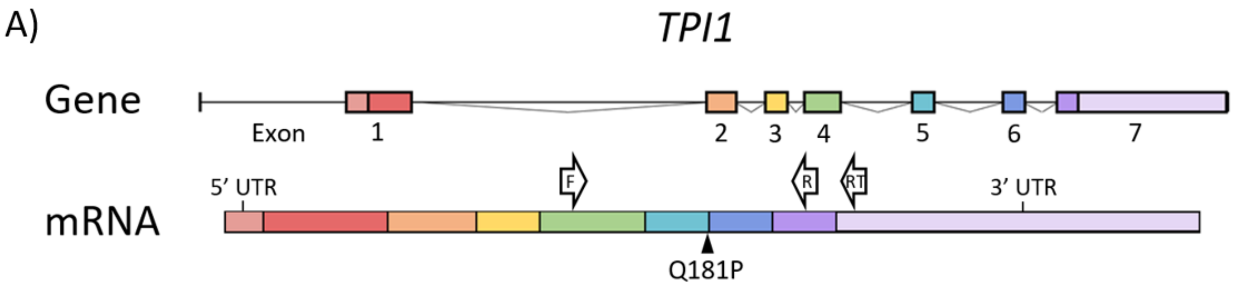

B)

	Splice Recognition Sequence		ASSP Score
Normal	CAG	GTAA	13.884
Q181P	CCG	GTAA	9.964
	Exon 5	Intron	

C)

Splicing Variant	Expected Length of PCR Product (bp)
Normal	351
Exon 5 Skipped	263

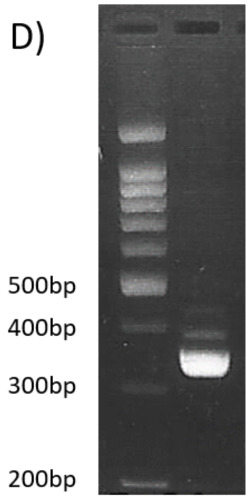

Fig. S1. *TPI1*^{Q181P} mutation alters the exon 5 splice donor sequence. A) Structure of *TPI1* gene and mRNA. Location of reverse transcription (RT) and PCR primers are noted. **B)** The splice recognition sequence is altered by *TPI*^{Q181P} per Alternative Splice Site Predictor (ASSP), where higher scores indicate a better splice site (Wang, M., & Marin, A. (2006) Characterization and prediction of alternative splice sites. Gene, 366(2), 219-227. doi:10.1016/j.gene.2005.07.015). **C)** *TPI*^{Q181P} was predicted to lead to skipping of exon 5 using varSEAK (JSI Medical Systems). The potential lengths of PCR products with or without exon 5 skipping are noted in the chart. **D)** RT-PCR was used to investigate splice variation of *TPI1*^{Q181P}. Whole RNA was isolated from *TPI*$^{WT/Q181P}$ human fibroblasts (Qiagen RNeasy Mini Kit catalog no. 74104). cDNA was generated (Invitrogen SuperScript III First-Strand Synthesis catalog no. 18080051) using a gene-specific reverse transcription primer. Exons 4-7 of *TPI1*^{Q181P} was PCR amplified (Invitrogen Platinum Taq DNA Polymerase High Fidelity catalog no. 11304011). PCR amplicons were visualized on a 3% agarose gel with ethidium bromide adjacent to a 100 bp ladder. The major product corresponds to a 351-base pair (bp) product, while there is an absence of a 263 bp product. These data suggest that exon 5 skipping is not prevalent.

Disease Models & Mechanisms • Supplementary information
